# Valproic Acid Induced Hyperammonemia in a Long Time Treated Patient

**DOI:** 10.1155/2016/6242314

**Published:** 2016-07-19

**Authors:** Rohit Aiyer, Margaret Seide, Robert G. Stern

**Affiliations:** Hofstra Northwell School of Medicine, Staten Island University Hospital, Staten Island, NY 10305, USA

## Abstract

We report a case of a patient who had been on long time valproic acid for treatment of bipolar affective disorder. While being an inpatient, serology ammonia level testing revealed a very high ammonia level despite being asymptomatic. Dual therapy of carnitine and lactulose was provided to the patient for treatment of the hyperammonemia. It should also be noted that, during this treatment, valproic acid was not stopped. Consequently, this case illustrates that patients can present asymptomatically despite very high ammonia levels and hyperammonemia can occur in chronic valproic acid despite not increasing the dose of the medication and psychiatrists do not need to discontinue valproic acid in the presence of elevated levels of ammonia if the patient shows no signs of encephalopathy or delirium.

## 1. Introduction

Valproic Acid (VPA) is a commonly used psychiatric drug for several disorders and is primarily used as a mood stabilizer and can also be combined with antipsychotics. Clinicians of all fields should be aware that toxicity of VPA can present clinically with lethargy, vomiting, and focal neurologic deficits as well as varying levels of consciousness, including coma in extreme cases [1]. As a result, it is important for physicians who prescribe VPA to their patients to closely monitor VPA level in both an outpatient and inpatient setting. There is evidence that these symptoms of VPA toxicity are secondary to the accumulation of VPA metabolites and increased levels of ammonia, hyperammonemia [1].

Hyperammonemia is generally defined as a serum level of ammonia greater than 40 *μ*mol/L [2]. It can be due to genetic conditions in newborns involving metabolism or liver disease, such as carnitine deficiency, malignancies, or portosystemic shunts. Elevated levels of ammonia can also be secondary to certain medications, such as 5-fluorouracil, salicylate, asparaginase, acetazolamide, diuretics, and VPA [2]. Levels above 200 *μ*mol/L are generally seen in neonate patients with inborn errors of metabolism, such as defects in the urea cycle and branch chain organic acidemia [2].

Therefore, we report a unique case of an asymptomatic presentation of a very high ammonia level (225 *μ*mol/L) in an adult patient that has been on chronic VPA treatment for bipolar affective disorder. Our study aimed to contribute to the literature and educate physicians in clinical practice with regard to management of hyperammonemia secondary to VPA therapy.

## 2. Case Report 

We present a 58-year-old female who presented to the emergency department with violent behavior and agitation. The patient had a history of bipolar affective disorder as per DSM-5/ICD-10 classification. According to medical records and collateral history from the patient's legal guardian, the patient was diagnosed with this mood disorder over 20 years ago due to repeated manic episodes which include irritability, labile effect, increased energy, and erratic behavior. Past medical history includes cellulitis, hypertension, hypothyroidism, and repeated urinary tract infections. The patient has a history of developmental delay with a diagnosis of intellectual disability. There was an absence of psychiatric illness in her family. During evaluation by the psychiatrist in the emergency room on admission, patient was clearly distressed, with her hair not groomed, and was slightly disheveled in her appearance. The patient was loud, yelling at the psychiatry and medical staff in the hospital, and was routinely uncooperative and belligerent. Patient was also clearly agitated which appeared in behaviors such as kicking and hitting the unit staff. There were no signs of psychosis, as the patient denied hallucinations in all sensory modalities and no delusions were elicited, and there was no evidence of disorganized thinking or paranoia. Her mood overall was angry and irritable, with a labile effect. At the time of examination, she was alert and oriented to person, place, and time. Due to her intellectual disability, she lacked judgment and had poor insight regarding her mental illness.

Patient was admitted with acute mania to the inpatient psychiatry unit and was continued on her home dose of valproic acid (VPA) at 1000 mg PO twice daily as well as continuing her other home medications (atorvastatin 10 mg PO at night, levothyroxine 175 micrograms PO once daily, and metoprolol 12.5 mg orally PO twice daily). It should be noted that the patient has been on VPA for over 10 years for treatment of her bipolar affective disorder, and her therapeutic levels and dosages are monitored by her outpatient psychiatrist. The high dose of her valproic acid is due to many years of outpatient psychiatry evaluation and titration of the dose to a level that allows for the patient to attain mood stability. Admission laboratory tests were all within reference range (CBC, BMP, and LFTs) and she had VPA level of 58 *μ*g/mL (therapeutic range: 50 to 100 *μ*g/mL). EKG showed slight sinus tachycardia, HR: 108.

During her admission, patient had episodes of severe agitation and her behavior was very unpredictable but was eventually controlled through psychotherapy and group therapy while being an inpatient. Patient was also encouraged to be compliant with her pharmacotherapy, particularly her mood stabilizer medication valproic acid. According to medical records, patient had a history of high ammonia levels on previous admissions, and therefore VPA levels and ammonia were monitored during the course of her hospital stay (Tables 1 and 2).

In the morning of day 51 of admission, patient was examined on the daily ward round. She was alert and oriented to person, place, and time and consciousness indicated that she was very much awake and aware of her surroundings, with no signs of altered consciousness. Patient was calm and cooperative during the psychiatric interview, with her appearance being well groomed. Her speech was of normal rate, rhythm, tone, and volume. There was no evidence of psychosis, and the patient appeared to be relatively euthymic with a full and congruent affect. Thought process was linear and thoughts were logical. Her judgment was still relatively poor and she had poor insight, but this was attributed in part to cognitive deficits. She had no neurologic deficits peripherally, and all cranial nerves were grossly intact. Lower extremity deconditioning secondary to lack of use had been an ongoing issue for over one year. Gait could not be assessed as she is nonambulatory but no abnormalities or deficits were present in her upper body chronically, and the patient had a Glasgow Coma Scale Score of 15 (Eyes: 4, Verbal: 5, and Motor: 6). Conclusively, patient elicited no clinical concerns from a neurological standpoint and therefore no clinical signs of encephalopathy or delirium.

Laboratory testing revealed an ammonia level of 225 *μ*mol/L (reference range: 10–40 *μ*mol/L) and VPA level of 117 (reference range: 50–125 *μ*g/mL). According to routine laboratory protocol, 0.5 mL of blood had been taken from the patient and transferred into a heparinized vacutainer and placed immediately on ice (for ammonia level). The specimen was transferred to the lab within 15 minutes of collection from the patient. Based on the Naranjo score of 9, it is reasonable to conclude that the elevated level of ammonia was secondary to VPA therapy [3]. Liver functions tests were within normal limits, congruent with her baseline. It was decided to treat the hyperammonemia with lactulose 20 grams orally twice a day as per standardized protocol, as she had 2 previous episodes of high levels of ammonia during her admission that resolved with the same dosing of lactulose (20 grams PO twice daily). Levocarnitine 990 grams PO three times daily was also started. More importantly, valproic acid was not discontinued since the patient reported no symptoms of valproic acid toxicity or hyperammonemia, as she was cognitively and neurologically intact.

After dual treatment with carnitine and lactulose, the patient's ammonia levels soon began to decline, from 225 *μ*mol/L to 163 *μ*mol/L to 27 *μ*mol/L within 2 weeks. The patient's dose of VPA did not change and she continued to receive this medication. During the course of these events, the patient did not develop any clinical signs or symptoms of hyperammonemia and remained stable neurologically and psychiatrically.

## 3. Discussion 

VPA is widely used in various neuropsychiatric and neurological conditions. It is considered to be safe with a wide therapeutic range [4]. Literature shows that high ammonia levels are elevated in 20% to 50% of patients prescribed VPA [5]. This is further confirmed in one study where ammonia levels were measured in 55 patients taking VPA. Twenty-nine of the 55 patients receiving VPA had elevated ammonia [6]. Another clinical study evaluated 12 patients on VPA treatment, and 83.3% (*n* = 10) of the sample had elevated ammonia levels. Three of the patients with elevated ammonia were noted to have possible effects from elevated ammonia. One had lethargy; the other had delirium and one had a nonspecific cognitive decline noted. The three with side effects had a mean ammonia level of 74.7 *μ*mol/L [7]. However, as per this case, the initial serum ammonia level of 225 *μ*mol/L correlated with no clinical symptoms on presentation.

For asymptomatic elevations in ammonia levels that persist, it is generally recommended to consider discontinuation of the medication [8]. Nevertheless, in this specific case, we illustrate that a patient who is on VPA acid chronically can continue the medication with concurrent treatment of carnitine and lactulose. This is important from a clinical standpoint, since stopping the VPA would have increased the risk of the patient relapsing into a manic state. It is also critical to note that hyperammonemia is usually of great concern when a patient has an increased dose of VPA, but, as per this case, it is evident that high levels of ammonia can occur even in stable dosing of the medication over many months. In all the cases discussed in one case series review, the patient's VPA was stopped and after a few days the patients' symptoms would resolve and ammonia levels would drop to normal range [2].

While majority of literature that reported on valproate induced hyperammonemic encephalopathy (VHE) is from neurology case studies, it should be noted that VHE was first reported in the field of psychiatry in 1995. A literature review demonstrated that, up until 2015, 36 cases of VHE have been reported from psychiatry reviews [4]. One example noted is a case report that described a 38-year-old woman with schizoaffective disorder and recent increase in VPA dosage that presented with somnolence and confusion and rapidly progressed to obtundation. She had normal liver function tests but serum ammonia level was severely elevated at 288 *μ*mol/L. Despite successful elimination of ammonia with hemodialysis she eventually developed fatal cerebral edema [9].

Structurally, VPA is a short, branched-chain fatty acid and is believed to complex with carnitine in a way that enhances renal excretion of carnitine [10]. The metabolism of VPA by mitochondrial oxidation produces propionyl-Co-A and valproyl-Co-A, which inhibit N-acetylglutamate synthetase which produces a depletion of N-acetylglutamate. This leads to the inhibition of CPS1 and therefore a decreased clearance of ammonia [11]. Carnitine supplementation (2–4 g/day) can be prescribed for both prevention and treatment of hyperammonemia secondary to VPA [12].

Consequently, it is evident that physicians should closely monitor ammonia levels even in patients that have been chronically treated with VPA with no increases in dosing, as the possibility for hyperammonemia should not be disregarded in this group of patients, as it occurs in 51% of psychiatric patients [13]. Clinical signs and symptoms are not always present, even in patients with relatively very high ammonia levels, and this should prompt physicians to monitor and treat it immediately in order to prevent clinical deterioration. Nevertheless, cessation of VPA is not always necessary in patients, as we have demonstrated that parallel administration of the medication to control the patient's bipolar affective disorder along with dual therapy of carnitine and lactulose can resolve the hyperammonemia.

## Figures and Tables

**Table 1:** Lactulose and carnitine administration and corresponding ammonia and valproic acid levels.

Admission day number	**1**	**6**	**9**	**10**	**12**	**13**	**20**	**34**
Ammonia (*μ*mol/L)			73	72	39			
Valproic acid level (*μ*g/mL)	78	60	72			50	64	77
Lactulose administered (grams)			20 grams q8h × 3 days					
Carnitine administered (mg)								

**Table 2:** Lactulose and carnitine administration and corresponding ammonia and valproic acid levels.

Admission day number	**41**	**51**	**54**	**57**	**60**	**65**
Ammonia (*µ*mol/L)	94	225	163	46	49	27
Valproic acid level (*μ*g/mL)	92	117	24	66	70	21
Lactulose administered (grams)	20 grams × 1 dose	20 grams q12h × 10 days				
Carnitine administered (mg)		990 milligrams q8h × 10 days				
